# Paving the Way for Fertilization: The Role of the Transmitting Tract

**DOI:** 10.3390/ijms22052603

**Published:** 2021-03-05

**Authors:** Ana Marta Pereira, Diana Moreira, Sílvia Coimbra, Simona Masiero

**Affiliations:** 1 Dipartimento di Bioscienze, Università Degli Studi di Milano, 20133 Milano, Italy; simona.masiero@unimi.it; 2 LAQV Requimte, Sustainable Chemistry, Universidade do Porto, 4169-007 Porto, Portugal; dianamoreira@fc.up.pt (D.M.); scoimbra@fc.up.pt (S.C.); 3 Faculdade de Ciências da Universidade do Porto, Departamento de Biologia, Universidade do Porto, rua do Campo Alegre, 4169-007 Porto, Portugal

**Keywords:** plant reproduction, reproductive tract, transmitting tissue, pollen tube growth, extracellular matrix, pollen tube guidance

## Abstract

Angiosperm reproduction relies on the precise growth of the pollen tube through different pistil tissues carrying two sperm cells into the ovules’ embryo sac, where they fuse with the egg and the central cell to accomplish double fertilization and ultimately initiate seed development. A network of intrinsic and tightly regulated communication and signaling cascades, which mediate continuous interactions between the pollen tube and the sporophytic and gametophytic female tissues, ensures the fast and meticulous growth of pollen tubes along the pistil, until it reaches the ovule embryo sac. Most of the pollen tube growth occurs in a specialized tissue—the transmitting tract—connecting the stigma, the style, and the ovary. This tissue is composed of highly secretory cells responsible for producing an extensive extracellular matrix. This multifaceted matrix is proposed to support and provide nutrition and adhesion for pollen tube growth and guidance. Insights pertaining to the mechanisms that underlie these processes remain sparse due to the difficulty of accessing and manipulating the female sporophytic tissues enclosed in the pistil. Here, we summarize the current knowledge on this key step of reproduction in flowering plants with special emphasis on the female transmitting tract tissue.

## 1. Introduction

Reproductive success is vital for seed formation in angiosperms. Seeds are the product of double fertilization, a process unique to angiosperms, where one sperm cell fuses with the egg cell to produce the embryo while a second sperm cell fuses with the central cell to form the endosperm, a nutritive tissue essential for the growth and development of the next generation [1].

Angiosperm reproduction begins when a pollen grain alights on the top of the stigmatic cells, where it adheres and hydrates, leading to the germination of the pollen tube (PT). The PT carries the two sperm cells through different female reproductive tissues—the stigma, the style, and the transmitting tract (TT)—and then it grows along the funiculus [2], reaching the ovule micropyle. After perceiving the embryo sac attraction signal, the PT moves into the ovule and penetrates the embryo sac through one of its synergids. After the PT bursts inside the receptive synergid, the sperm cells are released and fuse with the female gametes, initiating seed development [3,4,5,6]. The entrance of more than one PT into an embryo sac is tightly controlled, and late-arriving PTs’ entrance is prevented [7,8].

This reproductive process involves a rich network of intrinsic communications and signaling cascades that mediate interactions between the PT and the sporophytic and gametophytic female tissues, ensuring the fast and meticulous growth of the PT along the pistil tissues. A considerable part of PT growth and its interactions with female tissues occur in the TT, which plays a pivotal role in the successful completion of the fertilization process. PT guidance in the extracellular matrix of the TT pathway, from the stigma to the embryo sac, is known to occur without any influence from the target ovule and is defined as the “pre-ovular guidance” stage [9,10]. Actually, the TT constitutes a key innovation in flowering plants, being essential for their reproductive success. The advent of the TT is closely associated with the evolutionary origin of carpels and the process of postgenital fusion of the carpellary flanks [11].

This is not only a major step in angiosperm evolution, but it also offers a perfect opportunity for manipulation of pollination and PT growth, an invaluable tool in plant breeding programs. In Arabidopsis, the TT includes cylindrical cells and is composed of highly secretory cells characterized by an extensive extracellular matrix (ECM), forming a highway for the PTs to grow inside the pistil [12,13]. This multifaceted matrix is proposed to support and provide nutrition and adhesion for PT growth and guidance, being also a place of species recognition [14,15,16,17,18].

Despite the current advances in plant reproduction, there is certainly much more to know about the fundamental molecular mechanisms that regulate the interactions of the PTs within the female reproductive tract. Fascinating questions raised over the years about TT functions, namely the roles of the molecules present in this tissue, do not yet have answers. Here, we summarize the origin and formation of the TT and the most recent results on its important functions for the reproductive process.

## 2. The Transmitting Tract

The TT is a specialized structure, starting at the base of the stigma, running down from the apex to the base of the ovary [19]. The TT is one of the marginal tissues of the gynoecium, along with the septum, style, and stigma. It arises from a specialized tissue within the carpel margins with meristematic characteristics: the carpel margin meristem (CMM). In Arabidopsis, the gynoecium is formed by two congenitally fused carpels which elongate to form a cylindrical structure during its development. In the adaxial side of this cylindrical structure, from both sides, two internal ridges develop, the CMMs, towards the center of the gynoecium to eventually meet and fuse, forming the septum through postgenital fusion. On the borders of these CMMs, the placenta develops and gives rise to the ovule primordia and later to fully mature ovules. Only after the gynoecium fully closes do the TT and the style differentiate, generating a fully mature gynoecium [20,21,22].

**Figure 1:**
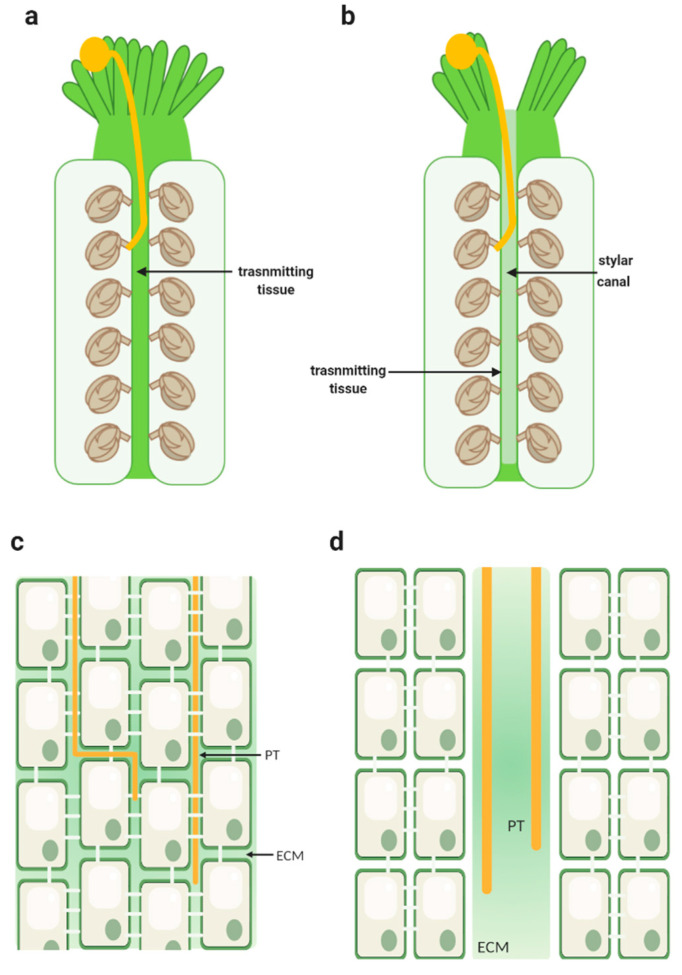
Schematic representation of a closed and an open style. (**a**) Longitudinal section of a closed style showing a continuous strand of transmitting tissue inside the pistil. A pollen grain is represented germinating in the stigmatic cells, with a pollen tube (in yellow) growing through the transmitting tract tissue towards the ovules. (**b**) Longitudinal section of an open style showing a continuous stylar canal lined with a secretory epidermis. A pollen grain is shown germinating in the stigmatic cells, with a pollen tube (in yellow) directing towards the ovules at the surface of the canal cells. (**c**) Longitudinal section of a part of the transmitting tissue from a closed style exhibiting the presence of substantial intercellular spaces filled with the extracellular matrix. Elongated cells are shown; they are connected through each other by the plasmodesmata in its transverse walls. Pollen tubes (PT) (shown in yellow) are displayed growing between the cells, in the extracellular matrix (ECM). (**d**) Longitudinal section of an open style showing the epidermal layer of secretory cells lining a canal filled with extracellular matrix (ECM); the pollen tube (PT) is yellow. Created with BioRender.com(accessed on 25 February 2021).

TT structure varies in the different types of styles; styles are classified as open or closed or semi-closed [12], depending on whether they are formed by a hollow channel or lumen, surrounded by a secretory epidermis (the transmitting tissue cells) that lines the canal producing mucilage (Figure 1b,d), such as in the lily, or formed by several layers of secretory tissue (the transmitting tissue), with a very small lumen but with large quantities of intercellular secretions (Figure 1a,c), such as in Arabidopsis. In both cases, narrow, elongated secretory cells, separated by the produced mucilage in the open styles and the ECM in the closed styles, form the transmitting tissue. Usually, these elongated cells communicate with each other, mostly through plasmodesmata localized in the transverse walls but not along its longitudinal walls. This type of cell organization presented by the TT is thought to be specially designed to facilitate PT growth along this tissue, providing them with a pathway of minimum resistance [18]. The presence of plasmodesmata is thought to be connected to the TT function in several ovary signal transduction pathways essential for pollen tube growth control and coordination [23]. Developmentally regulated programmed cell death (PCD) of the TT cells also contributes to the facilitation of PT growth. It seems to provide a smoother and more accommodating pathway for PTs to grow along this tissue and reach the ovules. Moreover, the deterioration of the TT cells is thought to be responsible for an increase in nutrient resource availability in the ECM to nurture the growing PTs. Furthermore, TT PCD is believed to have a protective role regarding fruit and seed set, by preventing the entrance of pathogens into the ovary, which would otherwise compromise fruit and seed development [24]. In Arabidopsis, TT PCD starts before anthesis and it seems to be enhanced by pollination [25], but in tobacco, it is triggered by pollination [24]; nevertheless, both studies pinpoint that this PCD is developmentally controlled and very specific for the TT.

Numerous studies have described the importance of a large set of transcription factors and regulators controlling TT formation and development, giving also important insights into its functions in reproduction. Given the origin and localization of the TT in the carpel, any mutation affecting genes whose products are essential for carpel fusion and development will affect TT development and formation too. Transcription regulators such as ETTIN, SPATULA, SEUSS, and AINTEGUMENTA [21,26,27] are just some examples of genes which are essential for the correct development of the gynoecium and consequently for TT development. Sessions and Zambryski [24] have shown that in *ettin* (auxin response transcription factor) mutant carpels, an everted TT develops in valve tissues and on regions with missing valves. Although misplaced, this TT seems to be functionally active since PTs can be seen growing along this tissue by aniline blue staining. SPATULA (SPT), a basic helix–loop–helix (bHLH) transcription factor, is fundamental for TT differentiation [21,28], since *spt* mutant carpels lack a TT and exhibit defects in the postgenital fusion of the septum. Moreover, the transcription adaptor SEUSS and the transcription factor AINTEGUMENTA play a role in TT formation; around 50% of the *seu-3 ant-1* gynoecia do not develop a TT [29]. Over the years, many other genes crucial for correct gynoecium development were described to be essential for TT establishment, such as ALCATRAZ [30] and INDEHISCENT [31].

Crawford and collaborators [25], in an elegant study, have shown that *NO TRANSMITTING TRACT (NTT)*, which encodes a C2H2/C2HC zinc finger transcription factor specifically expressed in the TT, is essential for its correct development. Arabidopsis *ntt* mutant plants lack a functional TT, showing impaired PT growth in the pistil due to defective ECM production and defective PCD. In the *ntt* mutant plants, the lack of a functional TT in the pistil is accompanied by a reduced staining for acidic polysaccharides by alcian blue in this region, indicating reduced arabinogalactan protein (AGP) content, considered essential for its functionality [32] and implicating them in this PCD process [19,25]. AGP involvement in this step must be verified by other methods, such as staining with the Yariv reagent or immunolocalization studies.

Besides NTT, several other genes contribute to the correct development of the TT, such as the redundant *HECATE (HEC1, HEC2, HEC3)* genes [33] and the closely related bHLH transcription factors, *HALF FILLED (HAF),* also known as *CESTA (CES)* and *BRASSINOSTEROID ENHANCED EXPRESSION1 (BEE1)* and *BEE3* [34]. HEC gene products modulate postgenital fusion of the septum and consequently TT development; *HEC* genes interact with *SPT* and together specify TT formation and development. As well as HEC transcription factors, HAF, BEE1, and BEE3 redundantly specify TT formation, being involved in ECM production, and besides this, they modulate cell death in the TT [34,35]. Recently, the MADS-box transcription factor SEEDSTICK was shown to act with NTT in the TT cell degradation process [36]. *STK* modulates TT development, acting together with *CES*, *BEE1,* and *BEE3* [37]. STK and CES influence PCD in the TT; in fact, the quadruple mutant *bee1 bee3 stk ces-4* transcriptome pinpoints that several genes whose products are involved in cell death are downregulated. Gene interaction studies have allowed Crawford and Yanofsky [34] to propose an elegant model describing the concerned action of these genes in reproductive tract development, which has now been suitably updated by Di Marzo and collaborators [37], including STK.

**Figure 2:**
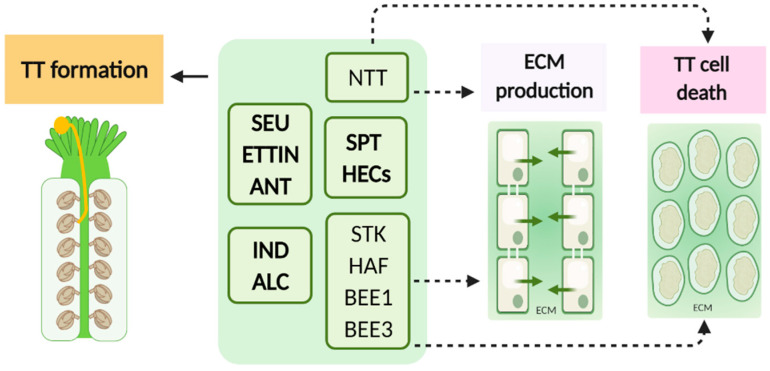
Key players involved in TT formation and development in *Arabidopsis thaliana*. So far, few main regulators involved in TT formation and development have been identified (NTT, NO TRANSMITTING TRACT; SPT, SPATULA; HEC1, 2, and 3, HECATE1, 2 and 3; STK, SEEDSTICK; HAF, HALF FILLED; BEE1 and BEE3, BRASSINOSTEROID ENHANCED EXPRESSION1 and 3) and a concrete genetic regulatory network has not been fully elucidated. NTT is necessary for TT differentiation. TT development is intimately related to correct carpel fusion and development; thus, several gene products that participate in such processes are also involved in TT formation (SEU, SEUSS; ETTIN; ANT, AINTEGUMENTA; IND, INDEHISCENT; ALC, ALCATRAZ). Proteins are grouped in different boxes according to the way they interact with each other. Created with BioRender.com(accessed on 25 February 2021).

As stated above, any gene which might be involved in carpel fusion might cause indirect defects in TT development. With this brief depiction of TT formation and development, we only show a small portion of the knowledge about this process, which is much more complex and finely regulated, involving the interaction of these regulatory gene networks with hormone signaling, such as auxin, cytokinin, and brassinosteroids. This has been extensively reviewed in [22,34,35]. The more recent work demonstrated the AHK cytokinin receptors expressed in the gynoecium to have redundant and specialized functions in this tissue of Arabidopsis [38]. Many genes have already been studied (Table 1 and Figure 2), but many more remain to be identified. Studies on this topic have contributed to a better understanding of the reproductive process, opening roads for others to study what goes on inside this tissue regarding PT growth, support, and guidance in greater detail, as will be discussed in the next section.

## 3. Key to the Highway: Pollen–Pistil Interactions in the TT

Several studies have confirmed that PTs grow more slowly, shorter, and less accurately in vitro than through the TT ECM, revealing its importance for PT growth [19,32]. Moreover, PTs grown in vitro are more efficient in targeting ovules if they first grow through a cut stigma and style [39,40,41]. Since the TT is part of the style, it is reasonable to assume that the PTs growing through the TT acquire the correct competency to respond to ovule attraction signals. Indeed, it has been proven that the transcriptome of PTs grown through a cut stigma and style is very different from the ones grown in vitro, revealing an entirely different set of activated genes involved in cell–cell communication processes [7,42,43,44]. This tissue seems to serve only one, but fundamental, purpose in the plant: to provide the finest environment for pollen tubes to grow along the pistil until reaching the embryo sacs embedded in the ovules and accomplish double fertilization [45].

In almost every species, the most striking feature of the TT is the massive deposition of ECM among its cells, whose nature and origin was very early on suggested to be a secretory product of the TT cells themselves [6,12]. The composition of this secretory substance, extensively studied in many angiosperms, is composed of carbohydrates, amino acids, glycoproteins, and glycolipids [14,15,16,46,47]. PTs either grow along the style canal filled with mucilage in the open styles or show an intercellular growth through the ECM of the closed styles [12]. The ECM is proposed to have a nourishing and supporting role for PT growth and is also the place where a large part of the pollen–pistil incompatibility interactions take place, an issue that has been extensively reviewed already [5,48]. Besides this, it is also the place where the PTs communicate with the female tissues by several signaling mechanisms in order to obtain guidance cues to grow in the direction of the ovules [49].

Cheung et al. [50] and Wu et al. [51,52] have confirmed the nutritive and adhesive role of the TT in PT growth by identifying AGPs in *Nicotiana tabacum,* designated as TTS (transmitting-tissue-specific), abundantly present in its ECM. TTS stimulate PT growth in vitro, attract PTs in semi-in vivo assays, and are essential for optimal PT growth in vivo. PTs can deglycosylate these glycoproteins while they grow along the TT, leading to the establishment of a gradient of increasing TTS glycosylation from the top to the bottom of the *Nicotiana* styles, suggesting that these glycoproteins may be a source of nutrients for PT growth and have a chemotropic guiding effect on growing PTs. The TT of Arabidopsis pistils and many other plant species is extremely rich in AGPs [6,7], which may be acting also as supportive or guiding factors for PT growth. AMOR, a 4-*O*-methyl-glucuronosyl arabinogalactan (AG) polysaccharide, was identified in *Torenia*; it is a sporophytic ovular factor that induces PT competency, meaning that PTs which have grown through a cut style become able to respond to female signals [44]. AGPs are strong candidates for the source of this AMOR factor, given their abundance in the female reproductive tract tissues and having this type of sugar. The molecular mechanisms underlying this signaling process are not yet known, and no AMOR molecules have been identified in Arabidopsis pistils, so far. Additionally, and more importantly, the PT growth through the style cannot be fully replaced by adding higher AMOR concentrations, signifying that other signals in the style, not yet identified, are necessary to induce PT competence. For instance, AGP presence in the reproductive tissues has been intensively studied over the years, and several important functions have been assigned to these glycoproteins, but it is still not known how they are integrated into the molecular mechanisms governing PT growth along the pistil tissues. AGPs are extremely glycosylated proteins predicted to be anchored to the plasma membrane by a GPI (glycosylphosphatidylinositol) anchor, attaching them to the extracellular face of the plasma membrane, and their sugar chains are proposed to be their active part in signaling mechanisms [53]. They are highly abundant in the female reproductive tract, all the way from the stigma until the ovule entrance, and their structural features strongly point to their active role as signaling molecules in these tissues [7]. In the *ntt* mutant plants, the reduced staining for alcian blue indicated also a reduced content in AGPs along the TT, putting forward a possible role for these glycoproteins in the TT PCD [19]. In fact, AGPs were, some years ago, suggested to be involved in PCD in plants [54]. Moreover, AGPs were recently described to act as Ca^2+^ capacitors by storing Ca^2+^ in the extracellular matrix, and the importance of this ion in reproduction is well described [55]. This capacity to bind Ca^2+^ seems to be conferred to AGP sugar chains. β-glucuronic acid (GlcA) residues, which are mainly found terminating the side chains of arabinogalactans (AGs) and which are also part of the AMOR factor described in *Torenia*, were shown to bind Ca^2+^ in vitro, in a pH-dependent manner [56]. By studying Arabidopsis mutants in AG β-glucuronyltransferases, responsible for glucuronidation of AG polysaccharides, Lopez-Hernandez et al. [56] revealed that a reduced content of GlcA on AGs led to deficiencies in plant development and in the spatiotemporal propagation of Ca^2+^ waves. This study has confirmed that the defects in plant development come from deficiencies in Ca^2+^ binding by poorly glucuronidated AGPs and, consequently, deficiencies in intracellular Ca^2+^ signaling. Given the high abundance of AGPs in the reproductive tissues and the special role of Ca^2+^ in this process, it will be extremely interesting to know what is happening at the reproductive level in these mutants.

Besides TTS, other players were discovered to have adhesion roles in PT growth along the TT, such as pectins and SCA [57]. SCA (stylar cysteine-rich adhesion) encodes a plant lipid transfer protein (LTP) which is secreted from the TT epidermis and is involved in adhesion-mediated PT guidance by forming an adhesive pectin matrix to guide PTs towards the ovules [58,59]. SCA must bind to pectin moieties to form an adhesive matrix between PT walls and the TT surface. In lily, chemocyanin, a small cell wall protein, was identified as a chemotropic compound from the stigma able to reorient PT growth in in vitro assays and in the stigma, an activity enhanced by the cooperation with SCA [60]. In *A. thaliana*, a plantacyanin gene sharing 86.8% similarity to lily, chemocyanin is abundantly present in the TT and is proposed to act in PT growth along this tissue [61]. Likewise in Arabidopsis, LTP5, a SCA-like molecule, was identified has having a role in PT tip growth, also by interacting with pectins [62]. SCA from the pistil is proposed to have both an adhesive and guiding role during PT growth, and pectin, a cell wall component highly abundant in the TT cell walls, plays an important role in this step of plant reproduction; VANGUARD1 (VGD) is a pectin methylesterase (PME) from PTs proposed to act during PT–pistil interactions by modifying TT cell walls. VGD1 is thought to enhance PT interaction with the transmitting tract. Demethylesterification of TT pectins by this PME may contribute to degradation and loosening of its cell wall. This may happen due to the release of protons during random homogalacturonan demethylesterification, which promotes the action of endopolygalacturonases contributing to the cell wall loosening and degradation, thus facilitating its interactions with the TT ECM [63,64]. Recently, a Zea mays female gene encoding a pistil-expressed pectin methylesterase 38 (PME38) homolog was identified and proposed to be involved in a mechanism for reproductive isolation in diverging plant populations of maize and teosintes, in which the PT cell wall is modified by the female tissues, consequently preventing continued PT growth in the TT and fertilization [65]. The γ-amino butyric acid (GABA) was also shown to have a role in PT growth, in Arabidopsis TT [66]. In vitro, GABA stimulates PT growth at lower concentrations and inhibits its elongation at higher concentrations. The gene *POLLEN ON PISTIL 2* (*POP2*) encodes a GABA transaminase which is involved in degrading GABA and establishing an increasing GABA gradient along the TT, believed to sustain PT guidance. GABA was proposed to mediate PT growth by modulation of putative Ca^2+-^permeable channels on the plasma membranes of tobacco PTs [67]. Ca^2+^ has been shown over the years to play vital roles during the reproductive process, particularly during PT growth [49]. Another pistil amino acid involved in regulating PT growth by modulating [Ca^2+^] in PTs is D-serine, which facilitates PT growth along the TT by inducing the Ca^2+^-permeable glutamate receptor-like channels (GLRs) at the PT tip plasma membranes [68]. Plant hormones may also play important roles during this step of PT growth. Vogler et al. [69] revealed that epibrassinolide, a brassinosteroid, enhances PT growth in vitro and suggests that cells from the TT provide brassinosteroid compounds to promote PT growth.

Finally, at some point, the PTs have to turn their growth direction towards the ovules and exit the TT fast lane. Although important PT components have been thoroughly described to act in PT change of direction [70], we do not know, yet, which kind of signal supports this change in growth direction. LUREs, a group of defensin-like polypeptides (DEFL), are known to act as PT attractants produced by the synergids in the ovules [71,72], but these are short-range signals and would not be capable of triggering PTs’ exit from the TT.

One of the topics which started to be tackled recently [36,37] and that will be surely further explored is the possible role of the TT regulators in affecting gene expression patterns of TT cell wall polysaccharides [73], since cell wall remodeling plays an essential role during PT growth along this tissue, being directly related to its nutritional, supportive, and guiding roles.

## 4. Concluding Remarks

The TT serves different and fundamental functions in PT growth, acting as a supportive, adhesive, nutritive, and guiding structure for PT growth, involving PCD, cell wall remodeling, and cell–cell communication processes. Although several genetic mutations affecting TT development have been discovered over the years, little information is available about the nature of PT growth-promoting ECM components along this important tissue. Insights pertaining to the nature of these factors and the mechanisms underlying the PT–pistil interaction processes remain sparse due to the difficulty of accessing and manipulating the female sporophytic tissues enclosed in the pistil.

**Figure 3:**
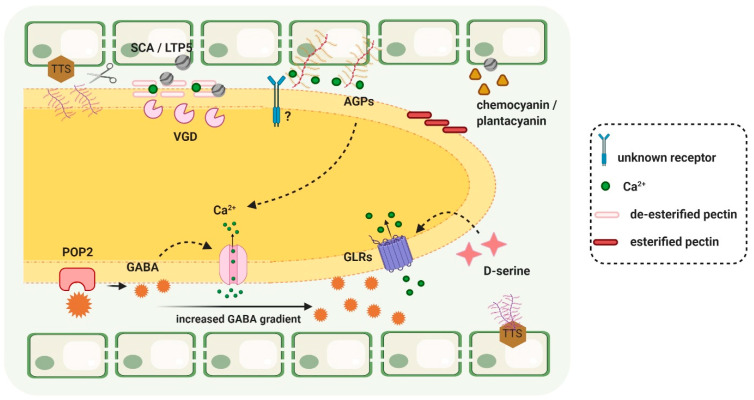
Main signaling molecules involved in the communication between pollen tube and transmitting tract during its growth in this tissue, towards the ovule. This image aims to focus mainly on the transmitting-tract-secreted signals, rather than on the signals generated by the pollen tube, well described in many reviews. TTS (transmitting-tissue-specific) is shown at the surface of the TT cells either still intact or being deglycosylated (scissors) by the growing PT. The glycosylated TTS is decreased after the arrival of the PTs in the TT, thereby establishing a gradient of increasing TTS glycosylation from the top to the bottom of the style. AGPs (arabinogalactan proteins) are also present in the TT cells and ECM and are thought to play essential roles in PT growth, by some signaling pathway which remains to be unraveled. AGPs are represented by the branched glycans acting as Ca^2+^ reservoirs (green dots), which are probably involved in Ca^2+^ signaling pathways in the PT. LTP (lipid transfer protein) represents the SCA, stylar cysteine-rich adhesion molecule, secreted by the TT cells, forming an adhesive matrix with pectins between the PT and the TT surface. Chemocyanin is also shown, which has a chemotropic guidance effect on PTs by acting together with SCA. VGD1 (VANGUARD1) is a pectin methylesterase (PME) from the PT, acting on pectin demethyesterification and leading to TT cell wall loosening to facilitate PT growth, possibly by coordinated action with endopolygalacturonases. A GABA (γ-amino butyric acid) gradient created by male POP2 (POLLEN ON PISTIL 2), a GABA transaminase, is thought to provide cues for PT growth along the TT. Besides this, GABA may modulate Ca^2+^ channels from the PT, influencing the dynamic extracellular and intracellular Ca^2+^ gradient essential for PT growth along the pistil tissues. Also involved in Ca^2+^ gradient changes is D-serine from the pistil tissues, which induces Ca^2+^ permeable GLRs (glutamate receptor-like channels) at the PT tip. Created with BioRender.com(accessed on 25 February 2021).

In recent years, research in the plant reproductive field has led to the discovery of innumerous players involved in the reproductive process. Most of the components discovered to be involved in pollen–pistil interactions are, nevertheless, molecular players present in the pollen and pollen tube, due to the facility inherent to its study. This was not the focus of the present review, and there are several reviews describing this in more detail, such as in Johnson et al. [49], Lopes et al. [74], and Mizuta and Higashiyama [10]. We aimed at gathering most of the information available on the female molecular players involved in the role of the TT for PT growth and guidance into the ovules (Figure 3), which greatly influences fertilization success. The importance of this step in the reproductive process demands further research. Identified transcription factors might serve as tools for future studies aiming at identifying the ECM TT components involved in pollen–pistil interactions. A better understanding of the molecular processes taking place during this crucial step of plant reproduction will not only be a great achievement in terms of advancing our knowledge of plant reproduction but will also enable us to eventually increase PT growth and fertilization efficiency and facilitate the generation of agronomically interesting interspecific hybrids.

## Figures and Tables

**Table 1:** List of the key players that are involved in TT formation and development in *Arabidopsis thaliana*.

Proteins	Type	Function	Expression Pattern	Phenotype of loss-of-function mutants	References
**NO TRANSMITING TRACT (NTT)**	C2H2/C2HC zinc finger transcription factor	Transmitting tract (TT) development; extracellular matrix (ECM) production and TT programed cell death (PCD)	TT	Non functional TT defective ECM production and defective PCD	[25]
**ETTIN (ETT)**	Auxin response transcription factor	Correct gynoecium formation	Gynoecium; valves; developing ovules	Everted TT develops in valve tissues & on regions with missing valves	[26]
**SPATULA (SPT)**	bHLH (basic helix-loop-helix) transcription factor	Acts redundantly with *ALC* to control development of carpel margin tissues	Developing carpel margin tissues	Unfused carpels; TT is absent; exhibits defects in post-genital fusion of the septum	[21,28]
**SEUSS (SEU)**	Transcriptional co-regulator	Development of the carpel margin meristem (meristematic structure located on the margins of the fused carpels and gives rise to ovules, septum, and TT)	Carpel margin meristem and ovules	Partial splitting of the gynoecial apex; *seu ant* double mutants exhibit ovule defects & 50% of the gynoecia do not develop a TT	[29]
**AINTEGUMENTA (ANT)**	Putative transcriptional regulator	Ovule development; TT development	Floral organs primordia & developing ovules.	Fail to produce ovules with integuments & functional female gametophyte; *seu ant* double mutants exhibit ovule defects & 50% of the gynoecia do not develop a TT	[29]
**ALCATRAZ (ALC)**	myc/bHLH transcription factor-like protein	Acts redundantly with *SPT* to control development of carpel margin tissues; fruit dehiscence	Developing carpel margin tissues; TT; stigma	Disruption of septum & gynoecium fusion; defects in TT development. Fruit dehiscence impaired	[30]
**INDEHISCENT (IND)**	bHLH DNA-binding superfamily protein	Valve margin development and silique dehiscence. TT development	Valve margins	Disruption of septum and gynoecium fusion. Fruits fail to open on maturity.	[31]
**HECATE 1, 2 and 3 (HEC1, HEC2, HEC3)**	bHLH transcription factors	Modulate post-genital fusion of the septum, and consequently TT development	Developing septum, TT and ovules	Infertility, defects in septum, TT and stigma development and impaired pollen tube growth	[33]
**HALF FILLED (HAF) / CESTA (CES)**	Transcription factor	Acts redundantly with BEE1 & BEE3 to specify reproductive tract tissues	Septum, TT and funiculus	In *haf bee1 bee3* ECM formation and PCD fail to occur within the TT; reduced pollen tube growth	[34]
**BRASSINOSTEROID ENHANCED EXPRESSION1 and 3 (BEE1, BEE3)**	bHLH transcription factor	Acts redundantly with HAF to specify reproductive tract tissues	BEE1: stigma & top of the style.BEE3 TT and style.:	In *haf bee1 bee3* ECM formation and PCD fail to occur within the TT; reduced pollen tube growth	[34]
**SEEDSTICK (STK)**	MADS-box transcription factor	Ovule identity; normal seed shedding; seed coat development; TT PCD	Septum and ovules	Fail to release seeds; Separation of abscission zone cells fails; fruits are shorter; abnormal positioning of seeds	[36,37]
